# Benign Intrapulmonary Schwannoma With High Uptake on Fluorodeoxyglucose-18 (FDG-18 PET) Presenting as a Pancoast Tumor

**DOI:** 10.7759/cureus.37788

**Published:** 2023-04-18

**Authors:** Sukhjinder Chauhan, Faraz Rahman, Gundip S Dhillon, Udanie Hewapathirana, Milliejoan Mongalo, Arnold Chung

**Affiliations:** 1 Internal Medicine, Mountainview Hospital, Las Vegas, USA; 2 Internal Medicine, Hospital Corporation of America (HCA) Mountainview, Las Vegas, USA; 3 Cardiothoracic Surgery, Mountainview Hospital, Las Vegas, USA

**Keywords:** benign schwannomas, flurodeoxyglycose-18, fdg pet scan, surgery, autonomic system, neurofibromas, schwannomas, posterior mediastinum, neurogenic tumors, mediastinal tumors

## Abstract

A 46-year-old female patient was diagnosed with a rare and benign intrapulmonary schwannoma, a neurogenic tumor that represents approximately 20% of adult mediastinal tumors, with schwannomas being the most common subtype. The patient was initially asymptomatic; however, after a period of four years, the patient presented with bilateral extremity edema, chronic venous stasis, elevated right ventricular systolic pressure, and a slightly enlarged inferior vena cava. These symptoms were caused by the lung tumor compressing intrathoracic structures.

This case highlights the need for early evaluation and proper management of neurogenic tumors to avoid serious symptoms and complications. It also emphasizes the importance of vigilant monitoring and prompt surgery to achieve the best outcome for patients with neurogenic tumors.

## Introduction

Approximately 20% of adult mediastinal tumors are neurogenic tumors [1]. These neurogenic tumors are typically located in the posterior mediastinum, a region rich in neurogenic structures, including the sympathetic chain, intercostal nerve, and vagus nerve [1-2]. Adult neurogenic tumors are classified based on the origin of the cell, such as schwannomas and neurofibromas arising from peripheral nerve sheaths, ganglioneuromas arising from the sympathetic chain, and paragangliomas arising from the parasympathetic chain. Schwannomas represent the most prevalent form of neurogenic tumors in the mediastinum [1-4]. Multimodal management by a team of specialists may be necessary to achieve optimal outcomes in treating these tumors.

## Case presentation

A 46-year-old Hispanic female with a history of a benign lung tumor, diagnosed four years prior, rheumatoid arthritis, and tobacco dependence presented to the hospital complaining of progressively worsening bilateral lower extremity edema, accompanied by an ulceration on the right medial malleolus. The patient was diagnosed with a primary intra-pulmonary schwannoma via biopsy of the lung mass approximately four years prior and referred to University of California, Los Angeles (UCLA) for surgical resection. However, she could not travel for the procedure due to financial constraints. At the time of her initial diagnosis four years ago, the patient was asymptomatic and reported no lower extremity edema.

On this admission, the patient reported the onset of bilateral lower extremity edema approximately six to seven months prior, which worsened over time. She first noted the appearance of a right medial malleolus ulcer about two months ago and was prescribed antibiotics by her primary care provider for possible cellulitis. However, the ulcer failed to heal despite antibiotic treatment. The patient reported experiencing mild dyspnea with exertion but denied any chest pain, syncope, shortness of breath at rest, hemoptysis, changes in vision, fever, or chills. Her current medications include dicyclomine, meloxicam, and ondansetron.

Upon admission, the patient was afebrile; vitals were remarkable, with a heart rate (HR) ranging in the 105-110s and blood pressure ranging around 150/80s mmHg. There were no signs of apparent respiratory distress, with normal oxygen saturation at mid-95%. On physical examination, there were diminished breath sounds in the anterior and posterior right upper lobes, with no crackles appreciated on lung auscultation. Jugular venous distension and prominent distension of neck veins on the right side were also noted. On peripheral vascular examination, bilateral lower extremity edema was observed, extending up to the knees, with grade 2 pitting edema and clean, non-purulent ulceration on the right medial malleolus.

**Figure 1:**
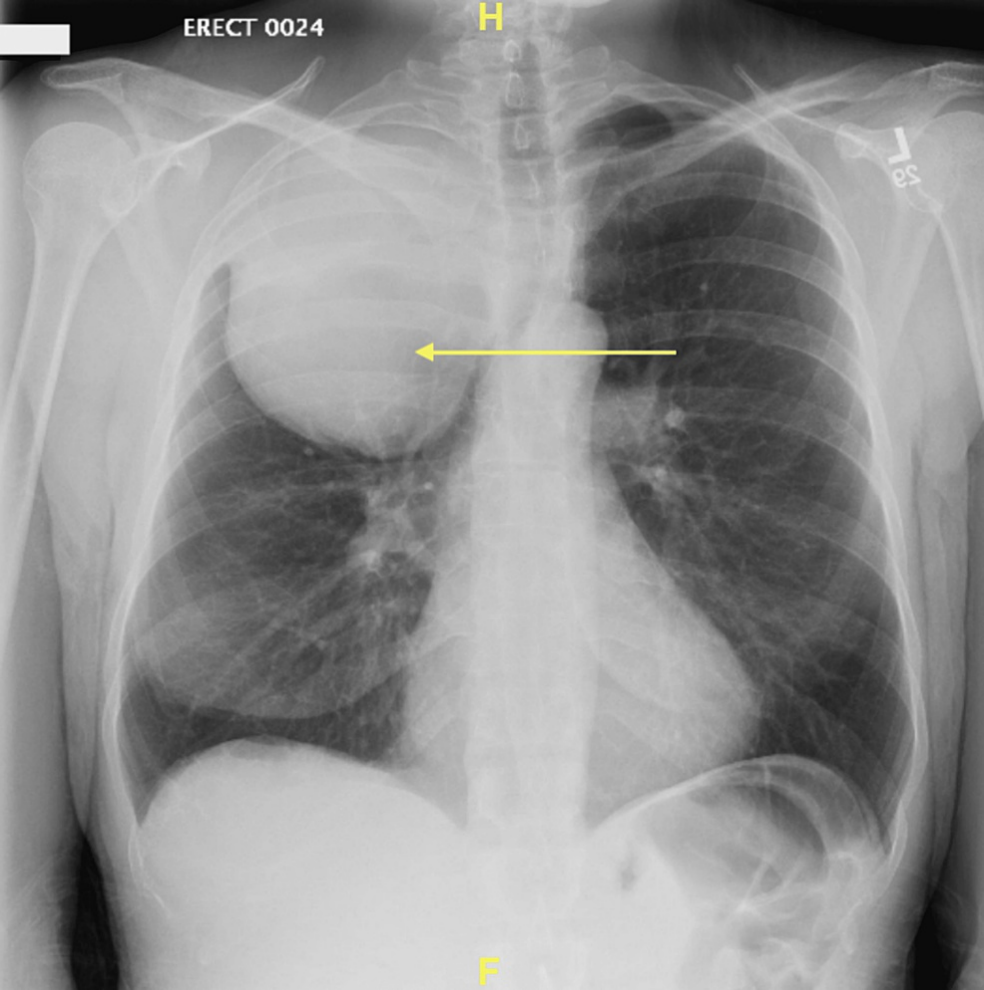
Chest X-ray demonstrating a circumscribed mass in the right upper thorax projecting over the right upper lobe.

The complete blood count (CBC) was remarkable for normocytic anemia, with a hemoglobin level of 9.6 g/dL. Otherwise, no abnormalities were noted. The comprehensive metabolic panel (CMP) results were within normal limits. The blood and wound cultures taken from the right medial malleolus ulcer were negative for growth. The chest X-ray (CXR) taken upon admission, as shown in Figure 1, revealed a circumscribed mass in the right upper thorax projecting over the right upper lobe. The lungs were otherwise clear, with no signs of remarkable pneumothorax or pleural effusion noted.

**Figure 2:**
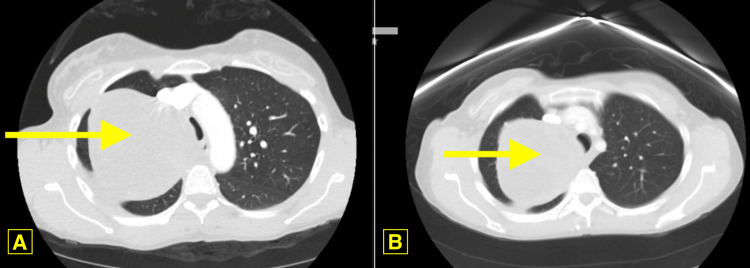
A computed tomography angiography of the chest (A), revealed a large, heterogeneous solid mass in the right apical region, measuring approximately 10.6 cm × 11.3 cm × 11.8 cm, that was contiguous with the pleural surface. The lung mass was found to have grown slightly since the previous CT chest, taken four years prior, which had measured to be 10.4 cm × 8.5 cm × 10.5 cm, as shown in the right panel (B).

A computed tomography (CT) angiography of the chest, shown in Figure 2A, revealed a large, heterogeneous solid mass in the right apical region, measuring approximately 10.6 cm × 11.3 cm × 11.8 cm and being contiguous with the pleural surface. The lung mass was found to have grown slightly since the previous CT chest, taken four years prior, which had measured to be 10.4 cm × 8.5 cm × 10.5 cm, as shown in the right panel of Figure 2B.

**Figure 3:**
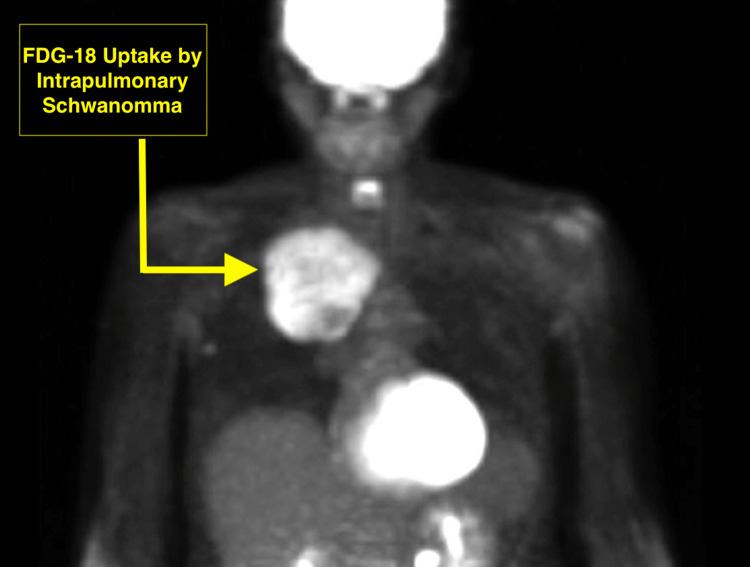
The fluorodeoxyglucose-18 positron emission tomography scan showed a standardized uptake value of 5.4, raising concerns about a possible hypermetabolic neoplasm.

At the initial presentation four years prior, a fluorodeoxyglucose-18 positron emission tomography (FDG-18 PET) scan was obtained, as shown in Figure 3, which revealed a standardized uptake value (SUV) of 5.4. This raised concerns about a possible hypermetabolic neoplasm.

**Figure 4:**
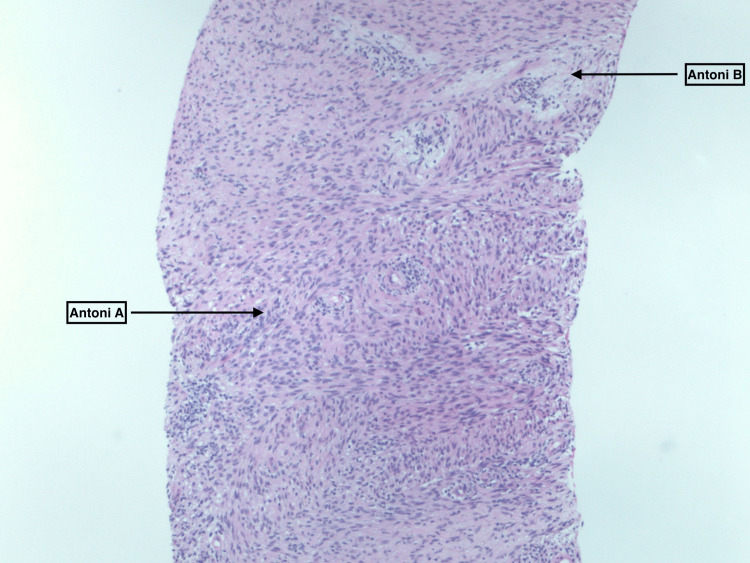
The microscopic histology of the mass demonstrating wavy nuclei in hypercellular regions known as Antoni A on the lower left side and hypocellular regions known as Antoni B on the upper right side of the image.

A CT-guided core needle biopsy performed four years prior showed that the mass tested positive for S-100, cytokeratin (CK), and AE1/AE and was negative for CD34, alpha-smooth muscle actin (SMA), and desmin on immunohistochemistry. The microscopic histology of the mass demonstrated wavy nuclei in hypercellular regions known as Antoni and hypocellular regions known as Antoni B, shown in Figure 4, consistent with a diagnosis of benign intrapulmonary schwannoma. 

The echocardiogram during this hospitalization revealed a left ventricular ejection fraction (LVEF) of 60-65% with a mild increase in left ventricular wall thickness. The right ventricle size was normal; however, the RSVP was mildly elevated at 38 mmHg (the normal range is 18-30 mmHg). Additionally, a mild dilation of the IVC was observed, measuring 2.8 cm (the normal range is 1.5-2.5 cm). The bilateral lower extremity venous Doppler study did not reveal the presence of deep vein thrombosis. A right ankle X-ray showed evidence of soft tissue swelling at the medial malleolus.

The cardiothoracic vascular surgery (CVTS) team was consulted to evaluate this lung tumor, which has increased in size and possibly compressed intrathoracic neurovascular structures. Upon thorough evaluation and complexity of the tumor, CVTS referred the patient to a tertiary care center for requiring complex surgical intervention. The patient was discharged with a recommendation to follow up with the UCLA medical team for ongoing management and monitoring. In addition, the patient was advised to use Unna boots to treat a right medial malleolus ulcer and to wear compression stockings to manage bilateral leg edema.

## Discussion

Schwannomas, also referred to as neurilemmomas, are the most frequently occurring nerve sheath tumors. They typically originate from the sensory root of an intercostal nerve or, in less frequent cases, from the phrenic or vagus nerve [3-4]. A retrospective study of 10 years, which reviewed 42 patients with thoracic neurogenic tumors, showed that 31 patients (74%) had tumors in the posterior mediastinum, 20 patients (48%) were diagnosed with Schwannoma, and six patients (14%) were diagnosed with malignant peripheral nerve sheath tumors (MPNST) [5]. Another study conducted by Takeda et al. reported similar findings, which included 146 patients diagnosed with thoracic neurogenic tumors; of the 93% (136 patients) found in the posterior mediastinum, schwannomas were the most common [6].

Intrapulmonary Schwannomas are a rare subtype of lung tumors that are typically benign and solitary. They can be located either centrally or peripherally within the lung tissue. These tumors' recurrence and malignant transformation rates are low [7-8]. The most prevalent presentation of intrapulmonary schwannomas includes thoracic pain, dorsal or intercostal neuralgia, respiratory symptoms, including cough and dyspnea, and Horner's syndrome. The extent of symptoms experienced depends on the tumor's size and location. The latter is particularly relevant in the case of Horner's syndrome, which is mainly observed in tumors situated in the superior-posterior region [9]. CT scans of the thorax are crucial in evaluating mediastinal neurogenic tumors, providing size, density, contrast enhancement, and relationships with the surrounding intrathoracic structures such as blood vessels and the spinal cord [10-11].

The metabolic characteristics of schwannomas on FDG-18 PET are poorly understood and cannot be used to differentiate benign from malignant forms [12]. Reports of increased activity on FDG-18 PET scans, or hypermetabolism, have been noted in follow-up tumor patients. However, this does not always indicate malignancy, as seen in schwannomas (benign) with an FDG-18 PET uptake outside the mediastinum. There is no relationship between FDG-18 uptake and the tumor's size or growth rate, typically measured by the Ki-67 index. Schwannomas often have high FDG-18 uptake, making it difficult to differentiate them from malignant peripheral nerve sheath tumors before biopsy or surgical intervention [13]. Histologically, benign schwannomas resemble normal Schwann cells with unique features, including wavy nuclei in hypercellular (Antoni A) and hypocellular (Antoni B) regions [5]. MPNSTs, on the other hand, show spindle-shaped cells with twisted nuclei and indistinct borders in a collagen-rich stroma. On the basis of immunochemistry, schwannoma cells show strong positivity for the S100 protein in both the nucleus and cytoplasm [14].

The primary treatment for intrapulmonary schwannomas is surgical resection, which is highly effective in achieving complete removal and has a low recurrence rate. Video thoracoscopy is a minimally invasive and safe approach for removing intrathoracic neurogenic tumors [15-16]. However, removing neurogenic tumors in the posterior mediastinum can pose a risk to intercostal nerves and blood vessels, mainly when the tumors are situated in the superior sulcus. Surgical resection carries a low risk of damaging important structures such as the pneumogastric and phrenic nerves, brachial plexus, stellate ganglion, subclavian and vertebral arteries, and thoracic duct during the surgical procedure [17].

Computed tomography angiography (CTA) is a widely adopted protocol for the preoperative evaluation of patients diagnosed with neurogenic tumors in the paravertebral sulci between T5 and T12 [17]. CTA aims to identify the segmental arteries supplying blood to the anterior spinal system and accurately locate the Adamkiewicz artery, which predominantly originates from either the left intercostal artery (75%) or the right intercostal artery (25%), between T9 and L1, and serves as the primary blood supply to the anterior spinal cord [18]. Despite these precautionary measures, cases of post-operative paraplegia have been reported in patients with neurogenic tumors in the T1-T5 region that may extend into the spinal canal [18]. Several neurosurgical techniques have been outlined to minimize these complications [19-21]. Neurosurgery evaluation and intervention are often required before the thoracic procedure and need the collaboration of multiple specialties to carry out the surgery successfully [19-21].

Our patient, initially diagnosed with benign intrapulmonary Schwannoma based on immunochemistry and microscopic histology of the mass (Figure 3), was asymptomatic for four years before presentation. Imaging revealed the tumor's location in the posterior mediastinum, where most neurogenic tumors are found [3-5]. The FDG-18 PET scan of our patient demonstrated an SUV of 5.4, indicating a hypermetabolic tumor. Subsequent CT scans showed a heterogeneous appearance and increased SUV in the right axillary lymph nodes. Schwannomas often exhibit high FDG-18 uptake, making it difficult to differentiate them from malignant tumors [12-13]. However, the relationship between FDG-18 uptake and the ability to differentiate benign or malignant forms of schwannomas is poorly understood and cannot be determined before biopsy or surgical intervention. Our patient's diagnosis was confirmed via a biopsy of the lung mass, which demonstrated the Antoni A vs. Antoni B format consistent with benign intrapulmonary Schwannoma [5,14].

Von Recklinghausen's disease (neurofibromatosis type 1), resulting from a Schwann cell development issue, is characterized by numerous neurofibromas on the skin and within internal organs. In a study of four cases of MPNST, 75% exhibited clinical signs of Von Recklinghausen's disease. The presence of widespread neurofibromas raises the likelihood of malignancy or future malignancy in posterior mediastinal tumors [22]. Our patient's physical examination showed no skin lesions indicative of neurofibromas. The biopsy results were consistent with benign Schwannoma, suggesting that the solitary intrapulmonary Schwannoma in the patient was not likely related to Von Recklinghausen's disease, which is commonly associated with malignant nerve sheath tumors [22]. Furthermore, the average age of patients with peripheral nerve sheath tumors was 43.54 years [6], comparable to our patient's age of 42 at the time of initial diagnosis.

Over the four years following the initial diagnosis, the size of the patient's intrapulmonary Schwannoma grew, possibly causing symptoms such as bilateral lower extremity edema and a moderately elevated RSVP of 38 mmHg, which may be a result of compression of intrathoracic neurovascular structures. Surgical resection is the preferred method of treating intrapulmonary schwannomas, which offers a high success rate for complete removal and minimal chance of relapse [15,16]. Considering the risks to neurovascular structures that may lead to post-operative paraplegia as described in references [19-21], our patient underwent a comprehensive evaluation by a team of cardiothoracic vascular surgeons and was referred to a tertiary care center for requiring complex surgery. The patient was also recommended to utilize compression stockings to manage the bilateral leg edema likely caused by the compression of the IVC by the intrapulmonary schwannoma and to follow conservative treatments for a right-side medial malleolus ulcer secondary to chronic venous stasis.

## Conclusions

Intrapulmonary Schwannomas are a rare type of lung tumor that is benign. The most common symptoms include thoracic pain, respiratory symptoms, and Horner's syndrome. CT scans of the thorax are crucial in evaluating mediastinal neurogenic tumors. Schwannomas, which are benign tumors, often exhibit high FDG-18 uptake, making it difficult to differentiate them from malignant neurogenic tumors. Hence, a biopsy is necessary to establish a definite diagnosis. Surgical resection remains the primary treatment modality for intrapulmonary schwannomas. Multidisciplinary collaboration between specialties such as neurosurgery and cardiothoracic surgery is essential to optimize surgical outcomes and minimize postoperative complications.
